# TABADO: "Evaluation of a smoking cessation program among Adolescents in Vocational Training Centers": Study protocol

**DOI:** 10.1186/1471-2458-9-411

**Published:** 2009-11-13

**Authors:** Laetitia Minary, Hervé Martini, Nathalie Wirth, Francine Thouvenot, Dovi-Stéphanie Acouetey, Yves Martinet, Abraham Bohadana, Denis Zmirou-Navier, François Alla

**Affiliations:** 1 Centres d'Investigation Clinique - Epidémiologie Clinique CIE 6, Institut National de la santé et de la Recherche Médicale, Nancy, France; 2 Epidémiologie et Evaluation Cliniques, Centre Hospitalier Universitaire Nancy, Nancy, France; 3 EA 4360 Apemac, Nancy-Université, Université Paul Verlaine Metz, Université Paris Descartes, Nancy, France; 4 Institut National de la santé et de la Recherche Médicale U 954, School of Medicine, Nancy, France; 5 Réseau Lorrain d'Alcoologie et des Dépendances Associées, Hôpital Villemin, Centre Hospitalier Universitaire Nancy, Nancy, France; 6 Service de pneumologie, Centre Hospitalier Universitaire Nancy, Nancy, France; 7 Tabacologue, Epinal, France; 8 EHESP School of Public Health, Rennes, France

## Abstract

**Background:**

Most of the efforts to reduce teenagers' tobacco addiction have focused on smoking prevention and little on smoking cessation. A smoking cessation program (TABADO study), associating pharmacologic and cognitive-behavioural strategy, on a particularly vulnerable population (vocational trainees), was developed. This study aims to evaluate the efficacy of the program which was offered to all smokers in a population aged 15 to 20 years in Vocational Training Centers (VTC). This paper presents the TABADO study protocol.

**Methods:**

The study is quasi-experimental, prospective, evaluative and comparative and takes place during the 2 years of vocational training. The final population will be composed of 2000 trainees entering a VTC in Lorraine, France, during the 2008-2009 period. The intervention group (1000 trainees) benefited from the TABADO program while no specific intervention took place in the "control" group (1000 trainees) other than the treatment and education services usually available. Our primary outcome will be the tobacco abstinence rate at 12 months.

**Discussion:**

If the program proves effective, it will be a new tool in the action against smoking in populations that have been seldom targeted until now. In addition, the approach could be expanded to other young subjects from socially disadvantaged backgrounds in the context of a public health policy against smoking among adolescents.

**Trial registration:**

Clinical trial identification number is NTC00973570.

## Background

Tobacco is a major cause of preventable death in the world. According to the World Health Organization (WHO), smoking kills almost 5 million people each year. By 2030, this number is expected to double [1]. Every day, about 100,000 young people become addicted to tobacco in the world [2]. Preventing tobacco addiction is a public health priority designated by the Framework Convention for Tobacco Control of the WHO, in force since February 27, 2005. The adolescent population is a major target of this endeavour.

Almost all smokers become regular smokers before age of 20 [3]. In France, the average age of smoking initiation is 13.4 years, and 33% of teenagers at age 16 smoke every day [4]. Most of these young smokers are already dependent on tobacco [5]. In one study, 86% of teenage smokers said they were addicted; almost one-quarter of smokers aged 17-18 years said they had tried to stop but only a small proportion (5%) had managed to quit smoking [4]. Teenagers, even if they never smoked on a daily basis, may encounter significant difficulties in their attempts to quit. They experience the same withdrawal symptoms as do adults and underestimate the difficulty of quitting smoking [6].

Despite this, efforts to fight against smoking in the teenage population have focused largely on programs to prevent rather than quit smoking. Systematic surveys have identified twice as many trials aimed at preventing smoking [7] as trials aiming at smoking cessation [8,9]. To date, the effectiveness of different methods of quitting has been well demonstrated for adults [10], but evidence is lacking for adolescents. Two meta-analyses of trials of strategies for smoking cessation among teenagers have recently been published [8,9]. They stressed the advantage of programs based on a psychosocial approach and implemented within schools. They also demonstrated the moderate effectiveness of withdrawal programs among teenagers. In both studies, the authors concluded the need for further research because the data were still too fragmentary and were based on research studies of varying quality. In addition, evidence is lacking on the effectiveness of the combination of the two strategies for adolescents -- pharmacological and cognitive-behavioural -- which has demonstrated a synergistic effect on adults [10].

Moreover, programs for helping with smoking withdrawal offered to young people were developed on the basis of programs for adults and do not sufficiently take into account the specific forms of tobacco addiction among the young and their needs [10]. The traditional model for adults of the progressive development of nicotine addiction (from the experimental stage to the casual stage, then daily consumption increasing in frequency until the stage of addiction) [11], does not seem to fit young smokers. Some adolescents may have experienced their first symptoms of dependence before smoking daily or began smoking daily upon experiencing their first symptoms [6]. Their smoking habit is also more influenced by their environment (family, friends, and media) [12].

Many criteria for successful smoking cessation programs for teenagers have been described in the scientific literature [8,9,13,14]. The first lecture delivered in the programs should be informative but not preachy. Accessibility of treatment programs, notably by implementing them within schools (integrating the programs during school hours), and their cost-free character (for consultation and nicotine replacement substances) are important for attracting teenagers to become involved in smoking cessation programs. Few teenagers spontaneously consult a physician for withdrawal assistance because they tend to underestimate the difficulty of quitting. The anonymity of a program as regards parents is also crucial in increasing the motivation of young people to participate. Inclusion in a program must be voluntary. Finally, programs to aid in breaking the tobacco habit should be based more specifically on the psycho-social skills of the individual and should take into account the psychological aspects involved in the process of smoking withdrawal by means of individual and collective coaching. The combination of all these factors would allow for maximizing the participation rate of teenage smokers in a program to aid smoking withdrawal.

On this basis, a smoking cessation program (TABADO, TABagisme chez les ADOlescents) was developed by a team of tobacco addiction specialist physicians and public health researchers. This program combines pharmacological therapy with cognitive-behavioural therapy in a vocational school setting and is intended for adolescents from 15 to 20 years. It comprises 3 stages: (i) a lecture about general information on tobacco addiction for all students, both smokers and non smokers; (ii) an individual consultation with a tobacco addiction specialist for smokers; (iii) then 4 group sessions of follow-up and cognitive-behavioural therapy for smokers. The program is implemented within the institution during school hours. Consultations with tobacco addiction specialists and the group sessions are free, as are any substances that may be prescribed and dispensed as nicotine replacements.

The program was designed to be implemented within a particularly vulnerable population: vocational trainees. It was meant to enhance equity by reducing inequality in the access to health education programmes. Vocational trainees generally come from the least privileged socio-economic groups. Their health behaviour is often unfavourable, and their access to treatment is more difficult than for students of the same age who are enrolled in general school or university education [15].

However, before expanding such programs, their efficacy must be assessed. Therefore, we implemented this evaluation study among the vocational training centers (VTCs) of the Lorraine region, north-east France. The objective of this paper is to present the TABADO study protocol.

## Methods

This was a quasi-experimental, prospective, evaluative, comparative study. It compared 2 groups: the "intervention" group benefited from the TABADO program while the "control" group, drawn from the same training curriculum but from different VTCs, did not benefit from any specific intervention other than treatments and education that are usually available. The main objective of this study was to evaluate the efficacy a smoking withdrawal program offered as part of a comprehensive approach to prevention to all smokers in a population of young students in VTCs.

The primary outcome is the rate of smoking abstinence after 12 months, measured on the basis of the statements of the whole sample, not only among volunteers. The rate of abstinence is defined by the number of smokers who quit smoking at D_0_+12 months relative to the total number of smokers at D_0_, where D_0 _is the day of programme inception. Abstinence is defined here as quitting smoking for at least 1 month.

The secondary outcomes are three: (i) overall prevalence of tobacco usage in the study institutions at 12 months; (ii) students' motivation to quit smoking (motivational score) and frequency of attempts to quit within the 12 months after the intervention; (iii) and rate of withdrawal from the program among the volunteers after 12 months. This article deals only with the primary outcome of the study.

### Setting and participants

This observational study will cover all students entering a participating VTC in the Lorraine region (eastern France, 2.3 million inhabitants, 51 centers, 16,500 vocational trainees), during the school years 2007-2008 and 2008-2009. Students from vocational training courses with at least 2 years duration will be eligible. The control group will consist of students in the same kinds of courses, in different VTCs. Thus, the control group will have a psycho-socio-economic-cultural profile similar to that of the experimental group, without the control subjects being involved by the participants in smoking cessation, which might have occurred if the control subjects had belonged to the same VTC [16].

### Inclusion criteria

All students, males and females, registered in the VTC for at least a 2-year training period, and ranging 15 to 20 years old, will be included in the study. Criteria for non inclusion are subjects with current serious psychiatric disorders or who may be susceptible to decompensation upon quitting smoking (major depression), or smokers who are already involved in an ongoing attempt to quit, with medical monitoring.

### Sample size calculation

According to the literature, we can assume a maximum 5% spontaneous withdrawal rate [17]. We hope to double this rate among participants in the experimental group after 1 year. This 10% rate is based on the assumption of an effective participation of 50% of smokers in the experimental group, with a smoking cessation rate of 15% among them and the regular 5% for non-volunteers in the experimental group. With two groups of the same size, an α two-sided risk of 5% and a power of 85%, the number of smokers per group must be 500. Thus, the total number of students to be included is 2000 (anticipating that the prevalence of smoking is 50%).

### Intervention

The TABADO intervention takes place in 3 stages. First, a general information session on tobacco consumption is delivered to all teenagers, both smokers and non smokers. For the former wishing to join the program, this session is followed by individualized consultations with a team of tobacco addiction physicians who visit the VTC and deliver personal advice and assistance in choosing nicotine replacement therapy, if needed. Volunteers then benefit from a small group approach, supervised by the same physicians, consisting of discussion sessions to share experiences, strengthen motivation, and prevent relapse. There will be 4 sessions in total, comprising individual counselling and work in groups spread over 3 months (taking into account the availability of the trainees because of their alternate-week training schedule [1 week of school time with 3 weeks of work in the field]). In cases of a counter-indication to nicotine replacement treatment, only the cognitive-behavioral approach will be offered with a motivational interview and advice on strategies for resisting the desire to smoke.

### Data collection (figure 1)

**Figure 1:**
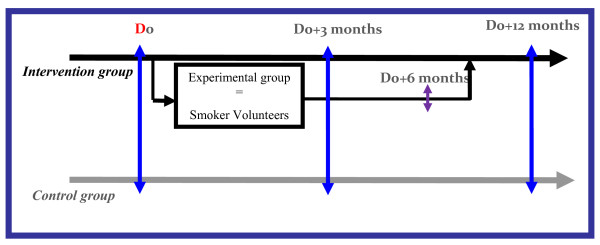
**General diagram of the TABADO study**.

#### Monitoring data for all students

An initial assessment questionnaire on the smoking status of each trainee, socio-demographic data, knowledge, attitude, and behaviour will be completed by all students during the first visit, which will take place in the same period for both groups. A final questionnaire to evaluate smoking status will be completed at D_0_+12 months. It will contain the items included in the initial questionnaire and items allowing for assessment of changes in the perception of the health risks of smoking among trainees. The questionnaire will also include questions to measure changes over time in tobacco consumption among smokers. Thus, it will allow an overall assessment of the effectiveness of the intervention among the target population.

#### Monitoring data for smokers who volunteer for the program only

The in-depth assessment of tobacco consumption of volunteers will be based on a questionnaire used by the tobacco addiction specialist and on the case report form. Monitoring over time of the volunteer's tobacco consumption will be provided by 4 questionnaires completed by volunteers at each visit with the tobacco addiction specialist. At 6 months after the start of the intervention, volunteers will complete a follow-up questionnaire.

Ethical approval for the trial was requested from Inserm (*Institut National de la Santé et de la Recherche Médicale *- National Health and Medical Research Institute). The protocol was submitted to the national scientific and ethical bodies involved (CCTIRS and CNIL), who gave their approval. Written consent from the subjects participating in the program was collected by one of us (LM), after subjects were given information about the program and the study, as well as their right to refuse to take part in the program or to leave it at any time. Two types of data and consent forms were used: one aimed at volunteer aged less than 18 and their legal representatives (parents or guardian) and the other addressed to adult volunteers [>18 years].

### Data analysis

The experimental and control groups will be compared for abstinence rates reported at 12 months after the intervention by use of multivariate logistic regression adjusting for predefined characteristics (age, sex, and training course). The analysis will be "in intention to treat". All the smokers from the institutions will be taken into account, and subjects lost to follow-up will be considered non-abstaining smokers.

## Discussion

Since 2002, nine reviews have been published concerning smoking cessation program among adolescents [8,9,18-24]. They all recommend further research and in particular call for studies that should include a follow-up greater than six-month; they also state that the primary outcome criteria should be validated by biochemical tests. The available data for evaluating pharmacological treatments are not conclusive, and produce contradictory results. Cognitive-behavioral approaches seem more promising but their efficacy is not yet proven. Moreover, combination of the two types of treatment, which has already proven its efficacy among adults [13], has not yet been validated among adolescents.

Therefore, according to Sussman's meta-analysis [9], the programs that showed a high smoking cessation rate were those that were offered in school settings and comprised treatments using motivation, cognitive-behavioral techniques and approaches based on social influences, and which contained at least 5 sessions. According to the review by Gervais [20] and a study by Kerjean [14], it seems necessary to initiate a specific program for adolescents that would be permanent, funded, and evaluated. Major factors boosting the efficacy of a program are, in particular, the confidentiality of the program, its voluntary nature, the fact that it is free and its accessibility. In addition, studies have highlighted specific traits in the population of trainees [15], which imply the need to test the feasibility and acceptability of such program geared towards such young pepole.

By combining these recommendations, the TABADO program should supply new evidence concerning the efficacy of smoking cessation in a population particularly vulnerable to tobacco addiction. TABADO requires significant financial and human resources (including the recruitment of physicians specialized in tobacco addiction), and its logistic is complex: the program must be adapted to the alternating pattern of the VTC school program (1 week at school and 2 weeks in the field).

The group nature of the intervention and the risk of "behavioural contamination" require cluster sampling, rather than individual sampling. Given the small number of institutions and their great heterogeneity in terms of training programs, we preferred a reasoned attribution rather than randomization, which might not yield comparable groups and would not be effective in this case [16]. Our intervention is a community-based intervention (i.e. interventions in which the unit of allocation to receive preventive regimen is an entire community [25]. In other words, judgment criteria are measured over the entire population rather than among volunteers only. The main risk is a dilution of the effect in the case of low participation [26].

Finally, we chose the rate of continuous abstinence after 1 year to ensure optimal conditions for evaluation: the period of 1 year is usually accepted as a general long-term monitoring standard [27].

## Conclusion

If the program proves effective, it will be a new tool in the action against smoking in populations that have been seldom targeted until now. In addition, the approach could be expanded to other young subjects from socially disadvantaged backgrounds in the context of a public health policy against smoking among adolescents.

## List of abbreviations

CHU: Centre hospitalier universitaire; CNIL: Commission Nationale de l'Informatique et des Libertés; ESCAPAD: Enquête sur la Santé et les Consommations lors de l'Appel de Préparation A la Défense; ESPAD: European School Survey on Alcohol and Other Drugs; HONC: Hooked On Nicotine Checklist; CI: Confidence interval; INCA: Institut National du Cancer; MILDT: Mission Interministérielle de Lutte contre les Drogues et la Toxicomanie; NVQ: National Vocational Qualification; OR: Odds Ratio.

## Competing interests

The authors declare that they have no competing interests.

## Authors' contributions

LM carried out the epidemiologic study, drafte the manuscript and will participate at the intervention for the data collection, and perform the statistical analysis. HM, NW and YM participated to the elaboration of the protocol. HM, NW, FT and SA will carry out the program as lecturers and tobacco addiction specialists. FA and DZN designed the study and drafted the manuscript. All authors read and approved the final manuscript.

## Pre-publication history

The pre-publication history for this paper can be accessed here:

http://www.biomedcentral.com/1471-2458/9/411/prepub

## References

[B1] World Health Organization The Tobacco Atlas 2009 http://whqlibdoc.who.int/publications/2002/9241562099.pdf

[B2] World Bank Publications The World Bank, Curbing the Epidemic, Governments and the Economics of Tobacco Control 1999 http://www1.worldbank.org/tobacco/reports.asp 10.1136/tc.8.2.196 PMC1759728 10478406

[B3] INSEE Enquête permanente sur les conditions de vie des ménages (partie variable): comportement vis à vis de la santé INSEE Première 2001

[B4] Beck F Legleye S Spilka S Les drogues à 17 ans, évolutions, contextes d'usage et prise de risque ESCAPAD 2003. Tendances 49 2006

[B5] Beck F Legleye S Spilka S Drogues à l'adolescence- Niveau et contextes d'usage de cannabis, alcool, tabac et autres drogues à 17-18 ans en France ESCAPAD 2003 2004

[B6] Difranza JR Rigotti NA McNeill AD Ockene JK Savageau JA St Cyr D Initial symptoms of nicotine dependence in adolescents Tob Control 2000 9 313 319 10982576 10.1136/tc.9.3.313 PMC1748379

[B7] Thomas R Perera R School-based programmes for preventing smoking Cochrane Database Syst Rev 2006 3 CD001293 16855966 10.1002/14651858.CD001293.pub2

[B8] Grimshaw GM Stanton A Tobacco cessation interventions for young people Cochrane Database Syst Rev 2006 CD003289 17054164 10.1002/14651858.CD003289.pub4

[B9] Sussman S Sun P Dent CW A meta-analysis of teen cigarette smoking cessation Health Psychol 2006 25 549 557 17014271 10.1037/0278-6133.25.5.549

[B10] HAS Stratégies thérapeutiques d'aide au sevrage tabagique Efficacité, efficience et prise en charge financière 2007

[B11] McNeill AD The development of dependence on smoking in children Br J Addict 1991 86 589 592 1859924 10.1111/j.1360-0443.1991.tb01813.x

[B12] Griesler PC Kandel DB Davies M Ethnic differences in predictors of initiation and persistence of adolescent cigarette smoking in the National Longitudinal Survey of Youth Nicotine Tob Res 2002 4 79 93 11906684 10.1080/14622200110103197

[B13] Agence Française de Sécurité Sanitaire des Produits de Santé Recommandations de bonne pratique Les stratégies thérapeutiques médicamenteuses et non médicamenteuses de l'aide à l'arrêt du tabac 2003

[B14] Kerjean J Stoebner-Delbarre A Adolescents and tobacco Journal de Pédiatrie et de Puériculture 2005 18 389 393 10.1016/j.jpp.2005.10.002

[B15] Kaminski A Nauerth A Pfefferle PI Health status and health behaviour of apprentices in the first year of apprenticeship - first results of a survey in vocational training schools in Bielefeld Gesundheitswesen 2008 70 38 46 18273762 10.1055/s-2007-1022528

[B16] Kemm J The limitations of 'evidence-based' public health J Eval Clin Pract 2006 12 319 324 16722916 10.1111/j.1365-2753.2006.00600.x

[B17] Chassin L Presson CC Sherman SJ Edwards DA The natural history of cigarette smoking: predicting young-adult smoking outcomes from adolescent smoking patterns Health Psychol 1990 9 701 716 2286181 10.1037/0278-6133.9.6.701

[B18] Backinger CL Fagan P Matthews E Grana R Adolescent and young adult tobacco prevention and cessation: current status and future directions Tob Control 2003 12 Suppl 4 IV46 IV53 14645940 10.1136/tc.12.suppl_4.iv46 PMC1766138

[B19] Garrison MM Christakis DA Ebel BE Wiehe SE Rivara FP Smoking cessation interventions for adolescents: a systematic review Am J Prev Med 2003 25 363 367 14580641 10.1016/S0749-3797(03)00213-7

[B20] Gervais A O'Loughlin J Dugas E Eisenberg M Wellman R Difranza JR Systematic Review of Randomized Controlled Trials of Youth Smoking Cessation Interventions Drogues, santé, société 2009 6 283 316

[B21] McDonald P Colwell B Backinger CL Husten C Maule CO Better practices for youth tobacco cessation: evidence of review panel Am J Health Behav 2003 27 Suppl 2 S144 S158 14521242 10.5993/ajhb.27.1.s2.5

[B22] Mermelstein R Teen smoking cessation Tob Control 2003 12 Suppl 1 i25 i34 12773783 10.1136/tc.12.suppl_1.i25 PMC1766088

[B23] Milton MH Maule CO Yee SL Youth tobacco cessation: a guide for making informed decisions 2004 Atlanta: U.S. Department of health and Human Services, Center for disease Control and Prevention 3 14

[B24] Sussman S Effects of sixty six adolescent tobacco use cessation trials and seventeen prospective studies of self-initiated quitting Tob Induc Dis 2002 1 35 81 19570247 10.1186/1617-9625-1-1-35 PMC2671530

[B25] Last JM A dictionary of epidemiology 1988 Oxford university press

[B26] Lindholm L Rosen M What is the "golden standard" for assessing population-based interventions?--problems of dilution bias J Epidemiol Community Health 2000 54 617 622 10890874 10.1136/jech.54.8.617 PMC1731718

[B27] U.S.Public Health Service Treating Tobacco Use and Dependence

